# Expedited CO_2_ respiration in people with Miltenberger erythrocyte phenotype GP.Mur

**DOI:** 10.1038/srep10327

**Published:** 2015-05-22

**Authors:** Kate Hsu, Mei-Shin Kuo, Ching-Che Yao, Ting-Ying Lee, Yi-Chun Chen, Han-Chih Cheng, Chia-Hao Lin, Tzung-Han Yu, Hui-Ju Lin

**Affiliations:** 1 Transfusion Medicine Laboratory, Mackay Memorial Hospital, Tamsui, Taiwan; 2 Department of Laboratory Medicine, Mackay Memorial Hospital, Taitung, Taiwan; 3 Department of Physical Education, National Taitung University, Taitung, Taiwan

## Abstract

In Southeast Asia, Miltenberger antigen subtype III (Mi.III; GP.Mur) is considered one of the most important red blood cell antigens in the field of transfusion medicine. Mi.III functions to promote erythrocyte band 3 expression and band 3-related HCO_3_^−^ transport, with implications in blood CO_2_ metabolism. Could Mi.III affect physiologic CO_2_ respiration in its carriers? Here, we conducted a human trial to study the impacts of Mi.III expression in respiration. We recruited 188 healthy, adult subjects for blood typing, band 3 measurements, and respiratory tests before and after exercise. The 3-minute step exercise test forced the demand for CO_2_ dissipation to rise. We found that immediately following exercise, Mi.III + subjects exhaled CO_2_ at greater rates than Miltenberger-negative subjects. Respiration rates were also higher for Mi.III + subjects immediately after exercise. Blood gas tests further revealed distinct blood CO_2_ responses post-exercise between Mi.III and non-Mi.III. In contrast, from measurements of heart rates, blood O_2_ saturation and lactate, Mi.III phenotype was found to be independent of one’s aerobic and anaerobic capacities. Thus, Mi.III expression supported physiologic CO_2_ respiration. Conceivably, Mi.III + people may have advantages in performing physically enduring activities.

Miltenberger antigens are glycophorin variants that belong to the complex MNS blood group system^1^,^2^. There are 11 glycophorin variants in the Miltenberger series (termed Mi.I to Mi.XI). From structural analyses, they presumably evolved from homologous gene recombination events between glycophorin A (GPA) and glycophorin B (GPB)^3^. Besides Mi.III, all the other Miltenberger antigens have extremely low occurrence frequencies worldwide, and are considered rare erythrocyte antigens in blood banking. The incidence of Mi.III is only 0.012% among Caucasians, and even lower among northern Chinese and Japanese^2^,^4^,^5^,^6^; its occurrence rates range between 2–6% in most parts of Taiwan and other Southeast Asian (SEA) regions^5^,^7^. GP.Mur is the protein entity of Mi.III phenotype. Because GP.Mur is highly antigenic, it is considered one of the most important red blood cell (RBC) antigens in blood banking in SEA^7^,^8^.

In human erythrocytes, GPA and band 3 are two most abundant membrane proteins, each with approximately 10^6^ copies in an average erythrocyte. Band 3, also known as anion exchanger-1 (AE1), facilitates HCO_3_^−^/Cl^−^ bidirectional transport across the red cell membrane. This anion exchange activity of band 3 plays an essential role in blood CO_2_ respiration^12^. Blood CO_2_ exists mostly in the form of HCO_3_^−^, and the conversion between CO_2_ and HCO_3_^−^ is mediated by carbonic anhydrase (CA) inside RBCs^12^. Band 3 serves as a gate on the RBC membrane for HCO_3_^−^ to enter or exit erythrocytes. Because the rate of HCO_3_^−^ conductance by band 3 is at least one order of magnitude slower than the rate of CA-mediated conversion, the anion transport activity of band 3, rather than the enzyme activity of carbonic anhydrase, is the rate-limiting factor for blood CO_2_ respiration^12^.

GPA serves as a chaperone that is responsible for the expression of band 3^13^,^14^,^15^,^16^. GPB, a structural homologue of GPA, expresses one-fifth the quantity of GPA on the red cell surface, and is functionally unclear^14^,^17^. In people bearing the Mi.III blood type, half or all of their GPB protein molecules on the RBC membrane are replaced by GP.Mur^18^. GP.Mur, encoded for Mi.III, is a naturally-occurred hybrid protein comprised of GPB and GPA. The sequence of the *GYP.Mur* gene contains a fragment of *GYPA* inserted in the sequence of *GYPB*^19^, and this unique glycophorin B-A-B hybrid structure is capable of promoting band 3 protein production and surface expression, just like GPA^18^. Our previous *ex vivo* study identified superior band 3-related functions (e.g. larger HCO_3_^−^/Cl^−^ transport capacities) in Mi.III + RBCs^18^,^20^. Since people with the Mi.III blood type express more band 3 on their red cells, their capacity of CO_2_ respiration were expected to be larger^18^,^20^.

The highest occurrence frequency of Mi.III worldwide is among the Taiwan Ami tribespeople (88%)^8^. Anecdotally, in Taiwan, Ami tribespeople are generally recognized for their athleticism and superior physical endurance, compared to people of other ethnic origins^21^. A disproportionally high percentage of the Amis are professional or elite athletes^22^,^23^. Ami people represent only 0.6–0.7% of the Taiwanese population, but over 50% of Taiwanese track athletes who had won international competitions were Amis^22^. Conceivably, these general impressions regarding the Ami people (athleticism and superior physical endurance) might be associated with the Miltenberger blood type that most of them bear.

The main goal of this study was to determine the impacts of Mi.III blood type in respiration through a large-scale human study. Faster CO_2_ expiration after a brief, physical challenge was observed in people with the Mi.III blood type. Ami tribespeople lacking the Mi.III phenotype did not have this respiratory advantage, indicating that the involvement of other tribal genes or factors is unlikely.

## Results

In this study, we recruited 266 non-athlete adults for questionnaire survey, physical assessment, blood tests (including Miltenberger phenotyping, band 3 measurements, and complete blood count), and a 3-minute step test (a standardized cardiorespiratory fitness test). The total sample number was down to 188, after excluding subjects who failed to complete the 3-minute exercise test, or who have one or more of the following conditions: hypertension, BMI > 30, cardiovascular disease(s), asthma, HBV/HCV hepatitis, joint/bone problems, cancer(s), or other major diseases. Among the selected, physically-healthy subjects, 39% are positive with the Mi.III blood type. Their initial physical assessment and RBC-relevant complete blood count data were summarized in Tables 1 & 2. Mi.III and the non-Mi.III participants were not distinguishable in most of the categories in these two Tables. On the other hand, Mi.III+subjects had slightly higher blood pressure than non-Miltenberger subjects in general (Table 1). Additionally, compared to non-Mi.III female, Mi.III+female subjects had in average smaller sizes of red cells (Table 2, MCV) and less hemoglobin per RBC (Table 2, MCH). The smaller MCH values in Mi.III+female were likely due to their smaller RBC sizes. The differences in MCV and MCH between Mi.III and non-Mi.III female however were not observed among the male subjects (Table 2). Because the differences in MCV and MCH were restricted to female, they were unlikely due to the expression of Mi.III. The gender differences in red cell sizes were probably related to other Ami-unique genetic factors, as many of our Mi.III+subjects are Ami tribespeople. These variations in blood pressure, MCV and MCH were still within the normal ranges

### Higher band 3 expression in Mi.III+subjects

**Figure 1:**
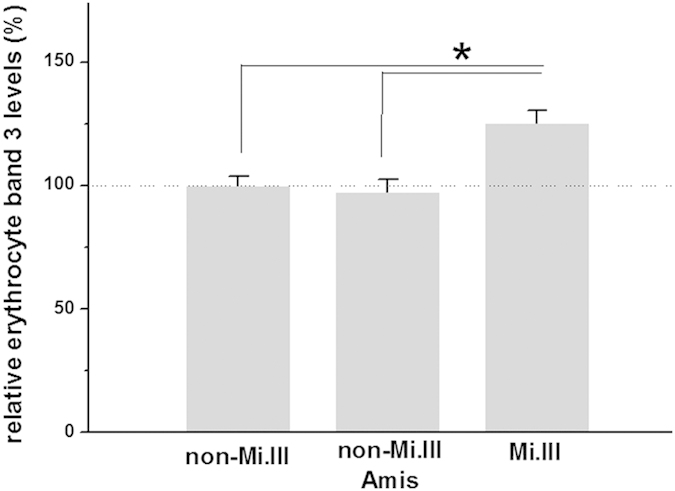
Mi.III+RBCs expressed more band 3 than Miltenberger-negative red cells. Relative band 3 expression was determined by DIDS labeling, and then normalized with respect to the average of non-Mi.III data (set 100%). Shown mean ± SEM. *p < 0.001.

Mi.III-encoded GP.Mur enhances the expression of erythrocyte band 3^18^. Here, we used a DIDS labeling method to verify band 3 expression levels in the red cells of these subjects. DIDS is a fluorescent compound that binds covalently and specifically to lysine-539 of band 3^24^. By DIDS labeling, we found that Mi.III+RBC samples expressed in average 25.2% more band 3 than the blood type-negative samples (Fig. 1), which is in agreement with our previous measurements by quantitative iTRAQ mass spectrometry, Western blot, and flow cytometry^18^,^25^. Because many of our Mi.III+subjects are Ami, Ami tribespeople who do not express Mi.III (“non-Mi.III Amis” in Fig. 1) were compared. As shown in Fig. 1, non-Mi.III Amis expressed significantly lower levels of band 3 than Mi.III+subjects. These results confirmed the direct association between Mi.III expression and higher band 3 levels, and excluded the possibility that other Ami-unique genes could also promote the expression of band 3.

### Expedited CO_2_ expiration following exercise in Mi.III+subjects

To determine the impacts of Mi.III in respiration, the subjects were asked to participate in a standardized 3-minute step test, and their cardiorespiratory parameters (end-tidal CO_2_, breathing rates, heart rates, and blood O_2_ saturation) were monitored before and immediately after the test. This 3-minute exercise test evaluates one’s cardiorespiratory fitness levels, and is backed up by a large population database on fitness collected by Taiwan National Council on Physical Fitness & Sports^28^.

**Figure 2:**
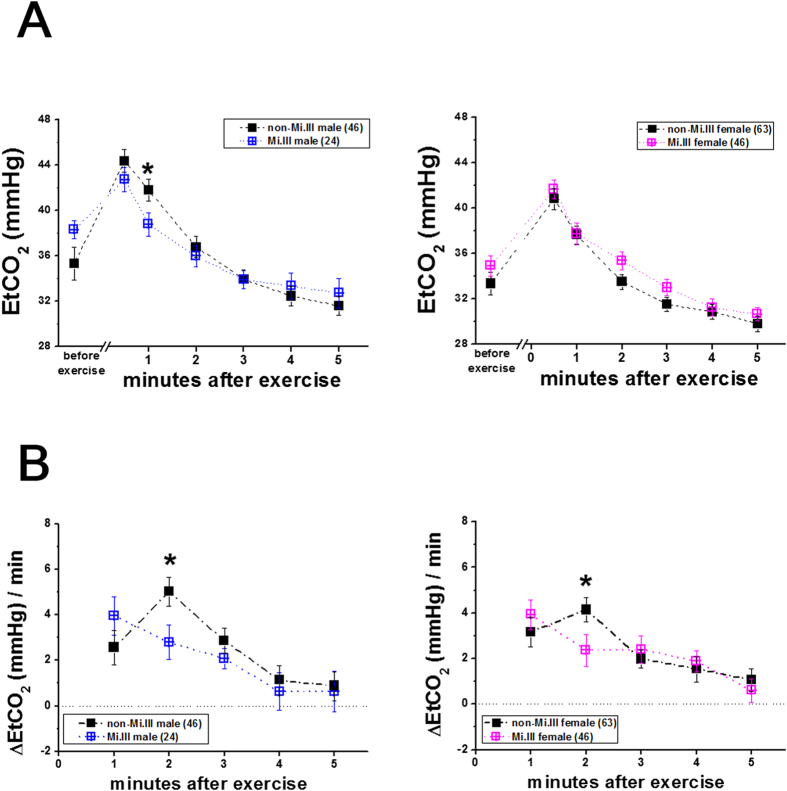
CO_2_ clearance immediately following exercise were faster for Mi.III+subjects than for subjects lacking Mi.III expression. Left: EtCO_2_ measurements in male participants; right: in female participants. (**A**) EtCO_2_ measurements for Mi.III (blue or pink color) and non-Mi.III (black). (**B**) Minute-to-minute changes of CO_2_ expiration (ΔEtCO_2_/min) were compared between Mi.III (blue/pink) and non-Mi.III (black). CO_2_ expiration in non-Mi.III participants remained substantial at the 2^nd^ minute following the step test, but had subdued in Mi.III+participants by the 2^nd^ minute (*p < 0.05). The numbers of the test subjects are indicated in parentheses. Shown mean ± SEM.

**Figure 3:**
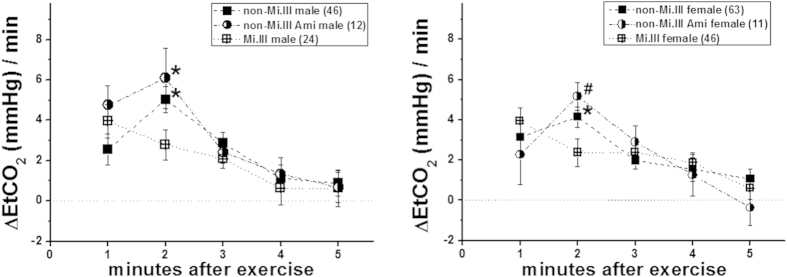
Expedited CO2 expiration following the step test was unique to Mi.III, and not to Miltenberger-negative Ami people The data for Mi.III+subjects are shown in cross symbols; for non-Mi.III subjects, in solid symbols; and for Miltenberger-negative Ami people, in half-solid symbols. Shown mean ± SEM. *p < 0.05. #*p* < 0.01.

**Figure 4:**
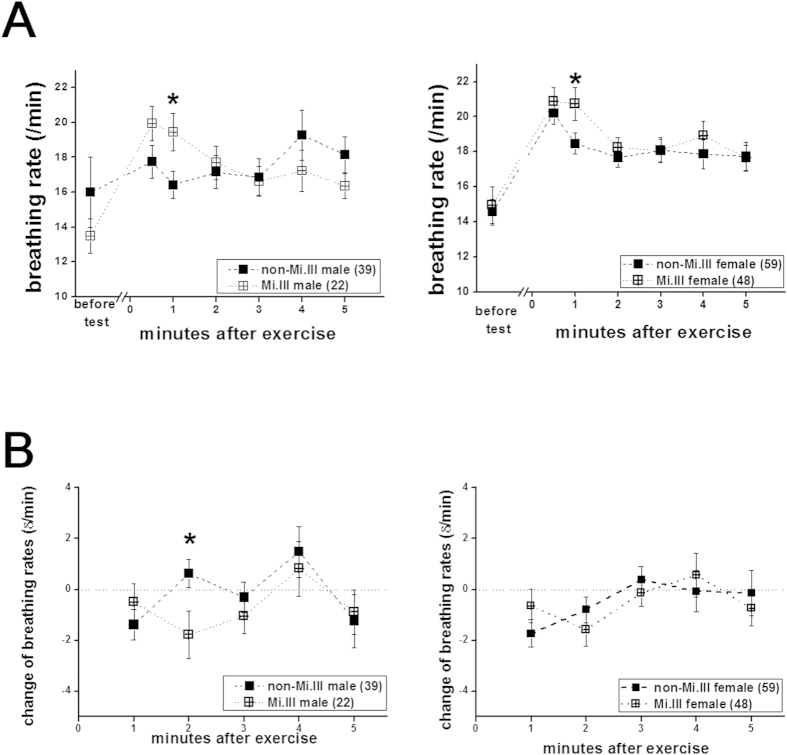
Breathing rates were also significantly higher in Mi.III+subjects immediately following the step test. Left: data from the male participants; right: from the female participants. (**A**) Breathing rates for Mi.III (cross symbols) and non-Mi.III (solid symbols) immediately before and after the exercise test. Faster respiration was observed within the first minute after exercise in Mi.III than in non-Mi.III subjects, regardless of their gender. The differences in breathing rates subdued at later measured time points. (**B**) Minute-to-minute changes of breathing rates. Shown mean ± SEM. *p < 0.05.

Among the physiological parameters measured, expired or end-tidal CO_2_ (Figs. 2,3) and respiration rates (Fig. 4) immediately following the step test were distinguishable between Mi.III and non-Mi.III. Mi.III+test participants generally exhaled CO_2_ faster right after the step test than Miltenberger-negative participants (Fig. 2). Because the amount of exhaled CO_2_ (as expressed in end-tidal CO_2_ or EtCO_2_ in the figures) varied greatly from individual to individual (data not shown), the minute-to minute changes of CO_2_ expiration (as expressed in ΔEtCO_2_/min) were calculated per individual, and then grouped for comparison (Fig. 2B). From the 30^th^ second to the 1^st^ minute post-exercise, there was 4.0 ± 0.8 mmHg drop of exhaled CO_2_ from Mi.III+male participants and 2.6 ± 0.8 mmHg drop of EtCO_2_ from non-Mi.III participants. From the 1^st^ to the 2^nd^ minute after exercise, the changes of CO_2_ expiration became 2.8 ± 0.8 mmHg for Mi.III+male participants, and 5.0 ± 0.6 mmHg for non-Mi.III male participants (Fig. 2B, left). That is, the rate changes of CO_2_ expiration for Mi.III+male had decreased by the 2^nd^ minute post-exercise, but that for Miltenberger-negative male participants remained substantial (Fig. 2B). Similar kinetic differences in CO_2_ expiration between Mi.III and non-Mi.III female were observed. From the 1^st^ to the 2^nd^ minute post-exercise, ΔEtCO_2_/min for Mi.III+female subjects had already dropped to 2.4 ± 0.7 mmHg, compared to 4.1 ± 0.5 mmHg at the same time interval for non-Mi.III female (Fig. 2B, right). For both male and female, the kinetic differences in CO_2_ expiration (ΔEtCO_2_/min) between Mi.III and non-Mi.III diminished by the 3^rd^ minute following exercise.

By comparing the differences in CO_2_ expiration kinetics between male and female, we also found slightly faster recovery in male than in female after the exercise challenge. At the 4^th^ minute post-exercise, ΔEtCO_2_/min have dropped to 0.6 ± 0.8 mmHg for Mi.III male and 1.1 ± 0.7 mmHg for non-Mi.III male, indicating plateauing CO_2_ expiration kinetics (Fig. 2B, left). When ΔEtCO_2_/min approaches zero, there would be no more change in the rate of CO_2_ expiration. On the other hand, for female subjects, ΔEtCO_2_/min were 1.9 ± 0.5 mmHg (Mi.III) and 1.5 ± 0.6 mmHg (non-Mi.III) at the 4^th^ minute post-exercise, and continued to drop (Fig. 2B, right).

To exclude other Ami-related factors than the Mi.III phenotype, we analyzed the data obtained from Ami subjects who lack Miltenberger expression. It is estimated that ~12% of Ami people do not express Mi.III blood type^8^. We found that the non-Mi.III Ami subjects showed significantly slower CO_2_ clearance than Mi.III+subjects, regardless of their gender (Fig. 3). Thus, expedited CO_2_ expiration following exercise was unique to people with Mi.III expression, and appeared unrelated to other Ami factors.

Besides CO_2_ expiration, breathing rates immediately following exercise were also significantly higher for Mi.III+subjects, regardless of their gender (Fig. 4). The differences in breathing rates between Mi.III and non-Mi.III diminished by the 2^nd^ minute following the test (Fig. 4A). We also calculated the minute-to-minute changes of breathing rates, and found that the changes between Mi.III and non-Mi.III male were significantly different during the interval from the 1^st^ to the 2^nd^ minute post-exercise (Fig. 4B, left). The kinetics of breathing rates for Mi.III+male had already dropped significantly by the 2^nd^ minute post-exercise (Fig. 4B, left). On the other hand, the female subjects, unlike the male, did not show significant differences in the kinetics of breathing rates between Mi.III and non-Mi.III (Fig. 4B, right); this is probably because the differences between Mi.III and non-Mi.III female were relatively small (Fig. 4A). Breathing frequencies are primarily controlled in response to changes of blood CO_2_ concentrations by the respiratory center in the central nerve system^33^. By comparing the changes of breathing rates and CO_2_ expiration (Figs. 2,4), their time course correlated well. By the 2^nd^ minute post-exercise, both changes in EtCO_2_ and in breathing rates decreased significantly in Mi.III+, as compared to the non-Mi.III (Figs. 2,4).

### Aerobic capacity not associated with Mi.III expression

The two other cardiorespiratory parameters measured before and immediately after exercise—heart rate and blood O_2_ saturation (SpO_2_), did not differ between Mi.III and non-Mi.III (Tables 3 & 4). Heart rate is an important indicator for one’s aerobic fitness^27^. One’s cardiorespiratory endurance index (CREI) score for each test participant could be calculated using three heart rate measurements after the step test, and be compared with the CREI norm charts published online by the Section of Physical Fitness & Sports of the Taiwan Sports Administration^28^. Mi.III and non-Mi.III participants exhibited similar levels of cardiorespiratory fitness (Table 3). Besides, the percent maximal heart rates (% max HR) at any time points measured post-exercise (from the 30^th^ second to the 5^th^ minute following the step test) also did not differ significantly between Mi.III and non-Mi.III (Table 3)^29^. So there appeared no associations between Mi.III expression and one’s cardiorespiratory fitness.

As for blood O_2_ saturation, there was a slight drop to 97.7–98.3% within 30 seconds post-exercise for all the subjects (Table 4). Their SpO_2_ levels recovered within 1 minute to the pre-test levels, and remained relatively the same from the 2^nd^ to the 5^th^ minute post-exercise in all 4 groups (Mi.III male/non-Mi.III male/Mi.III female/non-Mi.III female). There were also no significant variations in SpO_2_ observed at any time points before and after exercise between Mi.III and non-Mi.III (Table 4). In sum, these results suggested that Mi.III expression only expedited CO_2_ respiration (Fig. 2) and did not affect aerobic capacity (Tables 3 & 4).

### Distinct blood CO_2_ responses following exercise in Mi.III versus non-Mi.III

We further examined how Mi.III erythrocyte expression could affect one’s CO_2_ respiration by performing additional blood tests immediately before and after 3-minute step exercise on 30 male subjects. The tests included venous blood gas tests (CO_2_, pH, HCO_3_^-^ and base excess) and lactate measurements, and the results were summarized in Table 5. For all the subjects tested, 3-minute exercise resulted in small changes in venous CO_2_, HCO_3_^−^, and pH. This was expected, as the standardized 3-minute stepping test is considered moderate for healthy adults. For most of our test subjects, their heart rates remained below 60% HR_max_ immediately after exercise (Table 3). Anaerobic respiration in untrained people generally begin at 50–60% HR_max_^30^, so it was not surprising that there were very small increments of plasma lactate found immediately after the step test (Table 5).

We used analyses of covariate (ANCOVA) to test if any of the 5 parameters listed in Table 5 (CO_2_, pH, HCO_3_^−^, base excess, and lactate) were significantly affected by Mi.III expression following the exercise challenge. We found exercise-induced changes of venous CO_2_ to be significantly different between Mi.III and non-Mi.III subjects (*P < 0.05 by ANCOVA in Table 5). The other blood gas parameters and lactate were not affected by Mi.III expression, according to ANCOVA. On the other hand, by comparing the pre-exercise measurements between Mi.III and non-Mi.III subjects, or comparing their post-exercise blood measurements alone using *t*-test, we found no significant differences between Mi.III and non-Mi.III (Table 5).

**Figure 5:**
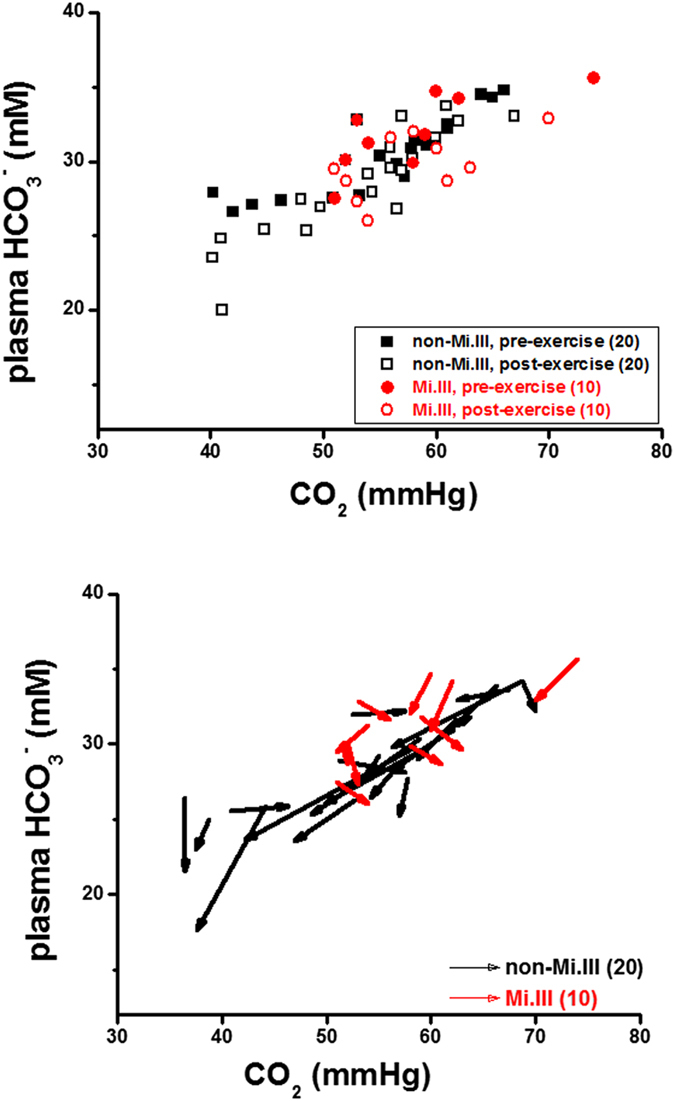
Mi.III and non-Mi.III people exhibited distinguishable blood CO_2_ responses following the step test. Top: A roughly direct correlation between venous CO_2_ and HCO_3_^−^ levels in Mi.III and non-Mi.III subjects alike. The concentrations of venous CO_2_ and HCO_3_^−^ immediately before 3-minute step test (shown in solid symbols) and after the test (empty symbols) were plotted for individual Mi.III (red symbols) and non-Mi.III subjects (black). Bottom: To further dissect the changes of venous CO_2_ and HCO_3_^−^, as well as their correlations, for each subject, his pre-exercise and post-exercise data points were connected and represented by a vector. Almost all the subjects, Mi.III and non-Mi.III alike, showed reduced HCO_3_^−^ levels after exercise. But noticeably, up to 70% of the non-Mi.III subjects had decreased blood CO_2_ levels after exercise, and only 40% of the Mi.III subjects showed slight decreases of blood CO_2_ following exercise. 50% of the Mi.III subjects showed slightly higher blood CO_2_ after exercise. The numbers of the test subjects per group are specified in parentheses.

We also found that a roughly direct correlation between venous CO_2_ and plasma HCO_3_^−^ levels (Fig. 5, top). This plot shows that despite the considerable variability in blood CO_2_/HCO_3_^−^ levels among individuals, blood CO_2_ levels were indiscriminately directly related to blood HCO_3_^−^ levels. To decipher the data, we plotted pre-exercise and post-exercise data points of all subjects again using vector representation (Fig. 5). Each vector composes of the pre-exercise and post-exercise measurements for a single participant. The beginning of a vector is one’s pre-exercise value, and the arrowhead is his post-exercise value. The length of a vector indicates the magnitude of change, and its directionality indicates how relevant or irrelevant the 2 parameters (represented by x- and y-axes) are.

From vector analyses (Fig. 5, bottom), we found that majority of the non-Mi.III subjects showed reduced levels of blood CO_2_ and HCO_3_^−^ following exercise. In comparison, half of the Mi.III subjects showed small increases of blood CO_2_, and the rest of the Mi.III subjects showed small decreases of blood CO_2_ after exercise. The magnitudes of blood CO_2_ changes among Mi.III subjects were generally smaller, compared to that in non-Mi.III (Fig. 5, bottom). The findings by vector representation supports the ANCOVA results (Table 5): exercise-induced venous CO_2_ responses were significantly different between Mi.III and non-Mi.III.

The results thus suggest that blood CO_2_/HCO_3_^−^ homeostasis in Mi.III+versus non-Mi.III people were distinguishable. Almost all the subjects showed slight decreases of plasma HCO_3_^−^ following exercise (Fig. 5 & Table 5). Though CO_2_ production increased during exercise, experimentally we and others observed reduced blood CO_2_ immediately after exercise in most non-Mi.III subjects^31^. It has been suggested that exercise-induced hyperventilation decreases body CO_2_ stores, in which bicarbonate is a main constituent^31^,^32^. This explains the reduction of blood CO_2_ and blood bicarbonate that we observed after the moderate exercise test (Fig. 5). Bicarbonate is also utilized to buffer excessive acidic metabolites generated during exercise, which would decrease the content of plasma HCO_3_^−^ following exercise, too^33^.

Conceptually, Mi.III RBCs express more AE1 on the cell surface than non-Mi.III RBCs (Fig. 1)^18^, and therefore Mi.III cells are expected to facilitate blood CO_2_/HCO_3_^−^ equilibrium more efficiently. Implied from our results (Fig. 5B), Mi.III expression might somehow affect blood CO_2_ sensing. Blood CO_2_ levels are primarily set by the respiratory center in the CNS^33^. Because Mi.III expression allows for faster reach of blood HCO_3_^−^ homeostasis and HCO_3_^−^/CO_2_ conversion, the respiratory center in Mi.III+people would need to cope with faster changes of blood CO_2_ levels at times. From what we found experimentally—the different venous 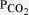 responses post-exercise between Mi.III and non-Mi.III (Fig. 5B & Table 5), conceivably the respiratory center in Mi.III+subjects could have higher tolerance for blood 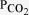. This also explains, at least in part, why our healthy Mi.III subjects generally had slightly higher blood pressure than the non-Mi.III subjects (Table 1).

### Similar degrees of anaerobic respiration induced by moderate exercise

**Figure 6:**
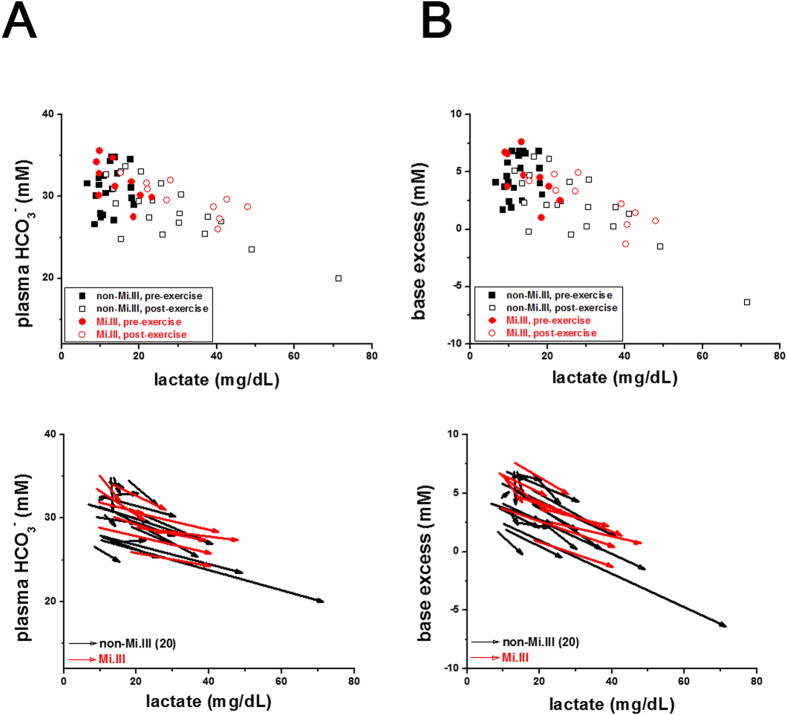
Mi.III and non-Mi.III people showed similar degrees of lactate production and base buffering following the exercise challenge. (**A**) roughly inverse correlation between plasma lactate and bicarbonate levels for all subjects before and after exercise. (**B**) A similar, inverse correlation between plasma lactate and base excess. Top: The concentrations immediately before 3-minute exercise (shown in solid symbols) and after exercise (in empty symbols) were plotted for individual Mi.III (red) and non-Mi.III (black) subjects. Bottom: The pre-exercise and the post-exercise data points per subject were connected and represented by a vector.

With the moderate exercise test, we observed very small accumulation of plasma lactate in virtually all the test subjects (Fig. 6 & Table 5). Blood lactate begins to accumulate when tissue oxygen is low and insufficient to provide energy for muscle contraction. Lactate acid is generated from pyruvate during anaerobic glycolysis to release anaerobic energy, and is a major metabolite of anaerobic respiration. Overproduction of lactic acid in muscle tissues (e.g. from strenuous exercise) may lower blood pH, which would then require buffering by metabolites like phosphocreatine, bicarbonate, and proteins^32^. From the vector analyses (Fig. 6), all the subjects, Mi.III and non-Mi.III alike, exhibited reduced plasma bicarbonate (or base excess) with increased lactate post-exercise. Statistical analyses using either *t*-test or ANCOVA found no significant differences in the lactate levels between Mi.III and non-Mi.III (Table 5). Thus, Mi.III expression does not seem to affect anaerobic respiration (Fig. 6).

## Discussion

Mi.III is the second most important erythrocyte blood type following ABO in the fields of transfusion medicine in Taiwan^5^,^7^. Mi.III-encoded protein, GP.Mur, structurally is a hybrid of glycophorin B and glycophorin A; it functionally resembles that of glycophorin A in facilitating band 3 expression on the erythrocyte membrane^18^,^25^. Higher expression of band 3 on Mi.III erythrocytes enlarges the capacity of HCO_3_^−^ permeation across the red cell membrane^18^. Since band 3 is the rate-limiting factor for blood CO_2_ metabolism^12^, Mi.III+people are expected to have more efficient CO_2_ respiration. Here, we showed experimentally that Mi.III+subjects exhibited faster CO_2_ respiration and clearance immediately following a physical challenge (Figs. 2, 3, 4). Exercise-induced venous CO_2_ responses were also distinguishable between Mi.III and non-Mi.III (Table 5 & Fig. 5). In contrast, Mi.III phenotype does not affect either aerobic parameters (i.e. heart rates and SpO_2_) (Tables 3,4) or anaerobic respiration (Table 5 & Fig. 6).

**Figure 7:**
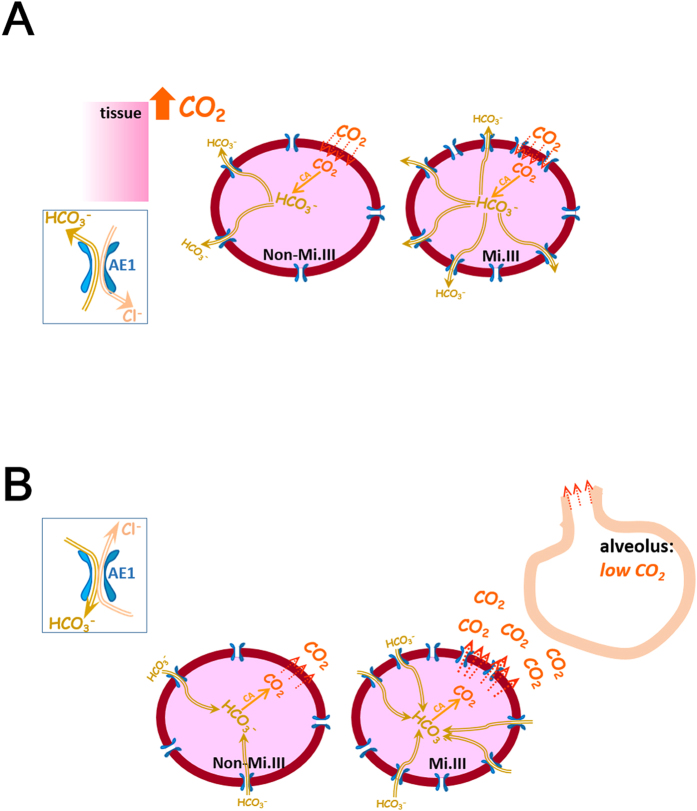
A proposed model on how CO_2_ expiration could be faster in people bearing the Mi.III blood type than those lacking this phenotype. (**A**) During exercise, there is increasing CO_2_ output, along with an increasing demand for ATP expenditure from contracting muscles. CO_2_ produced by muscle tissues enters erythrocytes primarily through passive diffusion (shown in red dotted arrows). Erythrocytes contain abundant carbonic anhydrase, which functions to greatly accelerate the chemical conversion between CO_2_ and HCO_3_^−^. Inside circulating red cells in this vicinity, CO_2_ is rapidly converted to HCO_3_^−^ by carbonic anhydrase. Much HCO_3_^−^ produced is passively transported out of RBCs via AE1 (shown as the blue gates embedded on the cell membrane). For every bicarbonate ion moving out of RBCs through AE1, a Cl^−^ ion is transported into RBCs to maintain electroneutrality across the cell membrane. The direction and magnitude of anion transport via AE1 follow the Donnan equilibrium, and thus this bi-directional anion transport does not consume energy. Mi.III RBCs express significantly more AE1 molecules on the membrane, allowing more efficient export of bicarbonate and faster reach of HCO_3_^−^ homeostasis across the cell membrane. (**B**) 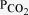 drops to below 40 mmHg in the lungs. The 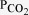 differences between lung alveoli and capillaries drive CO_2_ expiration. As most blood CO_2_ is present in the form of HCO_3_^−^, there is tremendous driving force for HCO_3_^−^ to be converted into CO_2_ in the lungs. Notably, the enzymatic activity of carbonic anhydrase to convert HCO_3_^−^ into CO_2_ is much faster than the rate of bicarbonate transport through AE1. Larger bicarbonate influxes into Mi.III RBCs that express more AE1, and consequently faster CO_2_ production and expiration are expected from Mi.III+individuals. The directionality of HCO_3_^−^/Cl^-^ fluxes through AE1 reverse from tissues to lungs, which is primarily driven by the changes of HCO_3_^−^ concentration gradients across the red cell membrane (as shown left in boxes). The diagram here is intended to illustrate the mechanism of how Mi.III expression could impact physiologic CO_2_ respiration, and is not drawn to scale.

To put in context with our current understanding of respiratory physiology, we propose a model to illustrate how CO_2_ expiration could be expedited especially in Mi.III+people following a physical challenge (Fig. 7). In lung alveoli (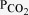 < 40 mmHg), the driving force to convert blood bicarbonate to CO_2_ is substantial. Since this conversion mainly takes place inside red cells, with larger bicarbonate influx capacities, Mi.III+RBCs in the lungs are expected to produce CO_2_ faster than non-Mi.III erythrocytes. We indeed observed faster CO_2_ expiration in people bearing the Mi.III blood type (Figs. 2,3). As Mi.III+RBCs circulate to the tissue vicinities, they are expected to facilitate blood CO_2_/HCO_3_^−^ homeostasis more efficiently than non-Mi.III cells, because of their higher AE1 expression (Fig. 7, top).

CO_2_ accumulation increases breathing discomfort and reduces one’s capability to endure physically^9^. Expedited CO_2_ expiration reduces the rate or the degree of CO_2_ accumulation in the body. Gorman *et al.* in their human study using insipiratory resistive loading found that lessening CO_2_ accumulation through ventilation prolongs endurance time^9^. Conceivably, when physically challenged, Mi.III+people with more efficient capabilities of CO_2_ respiration are expected to suffer less from CO_2_ accumulation or hypercapnia and be more capable of enduring physical stresses. Intriguingly, Ami tribespeople (88% with the Mi.III blood type) historically were recognized by the Japanese colonial government in Taiwan (1895–1945) to outperform other local ethnic groups in forced heavy labor^10^,^11^,^26^. One of the main reasons that the Japanese ruling officials in Taiwan at that time favored Ami tribespeople for labor-intensive tasks was Ami’s superior physical endurance, according to Yu-Chi Lai, a contemporary historian and anthropologist ethnic of Ami^34^.

To probe into the relations between Mi.III expression and physical endurance, we recently surveyed elite athlete students at the National Taiwan Sport University who had won medals in regional and/or national sports competitions, and found that 22% of them (16/72) have the Mi.III blood type. Moreover, up to a third of the track and field athletes (9/28) are Mi.III+. Notably, not all these Mi.III+elite athletes are ethnically Ami. About 11% of the non-Ami elite athletes have the Mi.III phenotype, which is at least double the frequency of Mi.III in the general Taiwanese population (2–6%)^5^,^7^. The Mi.III phenotype therefore might be associated with athleticism, at least in part through its support of physiologic CO_2_ respiration.

The HCO_3_^−^/Cl^-^ exchange capacity (permeability) of Mi.III+RBCs is expandable upon high HCO_3_^−^/CO_2_ stimulation^18^. People generate a lot of HCO_3_^−^/CO_2_ when they are physically stressed (e.g. during rigorous exercise). Our results here indicate that Mi.III expression in erythrocytes facilitate physiologic CO_2_ clearance. From this aspect, Mi.III-encoded GP.Mur protein could be perceived as a natural “de-stressor” through its support of band 3 expression. Expression of the Mi.III blood type is thus expected to be beneficial to one’s health.

## Materials & Methods

This human study aimed to test if GP.Mur phenotype could affect physiologic respiration through its support of band 3 expression.

### Ethics Statement

The study was carried out in accordance with the principles of the Declaration of Helsinki, and was approved by the Institutional Review Board (IRB) of Taiwan Mackay Memorial Hospital (MMH) (MMH-IRB registration number: 11MMHIS038). Written informed consent was obtained from all subjects.

### Study Design

Local adult subjects between 18–50 years of age were recruited at the three branches of MMHs in Taipei, Tamsui, and Taitung. To minimize interferences from ageing-related pulmonary function decline, people over 50 years old were refrained from participating in this study. The human trial included: (1) a questionnaire survey and initial physical assessments; (2) blood sample studies; (3) a 3-minute, step exercise test that accompanied respiratory physiological measurements, and/or blood tests. A quick prescreening of Mi.III was imposed to ensure that a high percentage of the test subjects have this special blood type. Prior to blood sample collection and the exercise test, each participant was first asked to fill out a questionnaire that surveys one’s lifestyle and health conditions. We also conducted initial physical assessments to exclude people who were unfit for the exercise test, i.e. those who were hypertensive (diastolic blood pressure >90 mmHg or/and systolic blood pressure >140 mmHg), or/and whose quiet heart rates were above 100.

### Blood tests and RBC phenotyping

Blood tests for all subjects participating in the trial included complete blood count (CBC), erythrocyte phenotyping including Mi.III, and a quantitative assessment of erythrocyte band 3 protein. The Mi.III RBC phenotype was serologically determined with anti-Mur, anti-Hil, anti-Anek, and anti-Mi^a^ antisera, each antiserum targeting a distinct epitope on GP.Mur protein. Mi.III phenotype was further confirmed by direct blood PCR, as described previously^35^. Additionally, venous CO_2_, bicarbonate, pH, base excess, and plasma lactate were tested in some of the male subjects who volunteered to have their blood withdrawn immediately before and after the 3-minute stepping test.

### Assessment of erythrocyte band 3 levels by DIDS labeling

RBC ghosts (membranes) were made by hypotonic rupture, followed by repeated washes to remove hemoglobin^18^. For each sample, RBC ghosts were mixed with an equal volume of 50 μM DIDS (Sigma-Aldrich, St. Louis, MO, USA), and incubated at 37 ^o^C for 30 minutes^36^. The fluorescent compound DIDS binds specifically to lysing-539 of band 3^24^. DIDS unbound to erythrocyte band 3 was then removed by PBS wash. DIDS labeling of band 3 was quantitated per sample by fluorescence emission using Varioskan^TM^ Flash Multimode Reader (Thermo Scientific, Waltham, MA, USA) at the emission wavelength of 450 nm (excitation at 350 nm). After fluorescence background subtraction, each DIDS emission reading was normalized with respect to the averaged value of all the Mi.III-negative samples, and expressed in percentile (the average of non-Mi.III data = 100%).

### The 3-minute step test and physiological measurements

The standardized 3-minute step test designed by the Taiwan National Council on Physical Fitness & Sports is a modification of the original Harvard test^37^. Like the Harvard step test, this standardized step test is to evaluate one’s cardiorespiratory fitness levels. Slightly different from the Harvard test or the YMCA step test^27^, the standardized step test used in Taiwan asks test subjects to step onto a 35-cm high sturdy bench at the rate of 24 steps (96 beats) per minute for continuous 3 minutes^37^. Heart rates were measured immediately following the test, and were used to assess one’s cardiorespiratory endurance index (CREI), as follows:


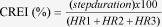


where step duration (in seconds) is 180 if the 3-minute test is completed. HR1, HR2, and HR3 refer to heart rates (beats/min) at the 1^st^, 2^nd^, and the 3^rd^ minute immediately after the step test.

The Department of Physical Education under Taiwan Ministry of Education conducts such standardized fitness tests annually on at least tens of thousands of people from 6–65 years of age. These test results were compiled in the norm charts of cardiorespiratory endurance indices for Taiwan populations^28^. In our study, besides heart rates, end-tidal CO_2_ (EtCO_2_), blood O_2_ saturation (SpO_2_), and respiration rates before and right after the step test were also measured using a portable oxi-capnography instrument (MD-667P; COMDEK Inc., New Taipei City, Taiwan).

ΔEtCO_2_/min Refers to the minute-to minute change of EtCO_2_ following the step test. ΔEtCO_2_/min at the N^th^ minute post-exercise was calculated by subtracting EtCO_2_ at the N^th^ minute from EtCO_2_ at the previous or the (N-1)^th^ minute post-exercise. Thus, ΔEtCO_2_/min represents the rate change of CO_2_ expiration.

### Statistical analyses

For most data, unless specified otherwise, the levels of significance (*p*) were determined by two-sample *t*-test. For analyses of blood test results obtained immediately before and after 3-minute step, separate analyses of covariance (ANCOVAs) were performed to examine the effects of Mi.III phenotype on each parameter. In ANCOVA, the fixed factor was the expression or the absence of Mi.III phenotype, pre-exercise measurements were used as covariate, and post-exercise measurements as dependable variables. A *p* value less than 0.05 was deemed significant.

## Author Contributions

K.H. and M.S.K. conceived the projects. M.S.K., Y.C.C., H.C.C., C.H.L., H.J.L. and T.H.Y. conducted the human trial. C.C.Y., H.J.L. and T.Y.L. carried out lab experiments and analyzed the data. K.H. wrote the paper.

## Additional Information

**How to cite this article**: Hsu, K. *et al.* Expedited CO2 respiration in people with Miltenberger erythrocyte phenotype GP.Mur. *Sci. Rep.* **5**, 10327; doi: 10.1038/srep10327 (2015).

## Figures and Tables

**Table 1:** Initial physical assessment for the healthy subjects participated in this study^5^.

**MALE**	**Number of healthy subjects**^1^	**Age (yrs)**	**Height (cm)**	**Weight (Kg)**	**BMI**^2^	**Ideal Body Weight (Kg)**^3^	**Exercise Frequencies (times/wk)**	**Blood Pressure (mmHg)**^4^	
							****	***Systolic***	***Diastolic***
***Non-Mi.III***	46	27.3 ± 9.1	172.6 ± 5.0	72.3 ± 11.0	24.3 ± 3.7	68.3 ± 4.5	2.0 ± 1.6	118.2 ± 13.7**	71.2 ± 11.0^**#**^
***Mi.III***	24	31.1 ± 10.0	174.0 ± 3.8	75.5 ± 12.1	25.0 ± 4.0	69.6 ± 3.5	2.4 ± 1.6	130.0 ± 13.0**	81.2 ± 11.6^**#**^

**FEMALE**									
***Non-Mi.III***	63	33.3 ± 10.6	158.5 ± 5.9	55.9 ± 7.0	22.3 ± 2.6	55.5 ± 5.3	1.7 ± 1.4	113.2 ± 11.4*	72.7 ± 9.9
***Mi.III***	46	35.3 ± 10.0	162.0 ± 4.7	60.2 ± 8.5	23.0 ± 3.4	58.7 ± 4.3	1.4 ± 1.3	121.5 ± 13.9*	73.7 ± 10.8

^1^Adult participants were deemed “healthy” subjects by the following criteria: BMI ≤30 (non-obese), and free from asthma, diabetes, hypertension, cardiovascular diseases, cancer, bone-and-joint problems, or major viral infection (HBV/HCV), and not in the Registry for Catastrophic Illness under the Taiwan National Health Insurance Program.

^2^BMI, body-mass index.

^3^Ideal body weight was calculated using the Devine equation^38^.

^4^Blood pressures were measured in quietness, prior to the exercise test. Statistically significant differences were found among subjects with the Mi.III phenotype and those without. **p < 0.005; ^*#*^*p* < 0.005; *p < 0.01.

^5^Data were presented in mean  ± S.D.

**Table 2:** A summary of the results of complete blood count (CBC) test for the subjects^3^.

**MALE (subject number)**	**RBC count (10**^6^**/μL)**	**Hb (g/dL)**	**HCT (%)**	**MCV**^1^ **(fL)**	**MCH**^2^ **(pg/cell)**	**MCHC (g/dL)**	**RDW (%)**	**RDW-SD (fL)**
***Non-Mi.III (46)***	5.1 ± 0.4	14.9 ± 0.9	45.4 ± 2.4	89.8 ± 6.7	29.5 ± 2.7	32.8 ± 0.8	13.5 ± 0.8	41.4 ± 3.2
***Mi.III (24)***	5.0 ± 0.3	14.7 ± 0.9	45.3 ± 3.4	90.6 ± 3.1	29.6 ± 1.3	32.7 ± 0.6	13.5 ± 0.6	41.6 ± 2.6
**FEMALE**								
***Non-Mi.III (63)***	4.4 ± 0.4	12.9 ± 1.0	38.9 ± 5.3	90.3 ± 7.4^**#**^	29.5 ± 2.9*	32.6 ± 0.7	14.2 ± 1.9	44.0 ± 4.1
***Mi.III (46)***	4.5 ± 0.4	12.5 ± 1.4	38.6 ± 3.7	85.2 ± 10.6^**#**^	27.8 ± 3.7*	32.3 ± 0.8	14.7 ± 2.8	42.6 ± 3.4

^1^MCV (mean corpuscular volume), the average volume of a RBC in fL, was significantly different among Mi.III+ and Mi.III-negative female subjects (^*#*^*p* < 0.01), but not among the male subjects.

^2^MCH (mean corpuscular hemoglobin), the average quantity of hemoglobin in a RBC, had significant variations between Mi.III and non-Mi.III in female (*p < 0.05), but not in male.

^3^Data were presented in mean ± S.D.

**Table 3:** Heart rates (HR) before and immediately following the stepping exercise test^2^.

**MALE**	**Quiet HR (/min)**	**Cardio-respiratory Endurance Index (%)**	**Predicted Max HR**^1^ **(/min)**	**HR (% max HR) at 30**^**th**^ **sec post-test**	**HR (% max HR) at 1st min post-test**	**HR (% max HR) at 2**^**nd**^ **min post-test**	**HR (% max HR) at 3**^**rd**^ **min post-test**	**HR (% max HR) at 4**^**th**^ **min post-test**	**HR (% max HR) at 5**^**th**^ **min post-test**
***Non-Mi.III (46)***	74.7 ± 14.7	65.9 ± 18.8	228.0 ± 6.2	119.0 ± 17.8	104.8 ± 18.5	94.5 ± 18.7	91.0 ± 17.7	91.8 ± 16.5	90.7 ± 16.1
				(52.4 ± 7.9)	(46.2 ± 8.2)	(41.7 ± 8.2)	(40.2 ± 7.7)	(40.2 ± 7.2)	(40.0 ± 7.1)
***Mi.III (24)***	80.5 ± 12.9	60.9 ± 10.1	231.2 ± 7.5	121.2 ± 14.8	108.8 ± 15.1	99.9 ± 17.9	94.3 ± 17.6	97.1 ± 17.4	94.9 ± 15.5
				(52.4 ± 6.5)	(47.3 ± 6.5)	(43.4 ± 8.1)	(41.0 ± 7.9)	(42.4 ± 7.7)	(41.4 ± 6.9)
**FEMALE**									
***Non-Mi.III (63)***	78.3 ± 10.7	57.5 ± 9.6	231.9 ± 7.1	135.0 ± 18.7	118.7 ± 18.7	105.2 ± 16.7	98.5 ± 14.9	95.0 ± 13.6	93.7 ± 13.9
				(58.3 ± 8.5)	(51.3 ± 8.3)	(45.1 ± 7.4)	(42.2 ± 6.5)	(40.7 ± 6.0)	(40.2 ± 6.1)
***Mi.III (46)***	80.0 ± 12.1	55.3 ± 8.2	232.6 ± 7.0	135.8 ± 16.1	121.4 ± 18.4	108.6 ± 15.9	102.8 ± 14.2	99.3 ± 14.3	97.4 ± 13.8
				(59.0 ± 7.4)	(52.7 ± 8.3)	(46.5 ± 7.4)	(44.1 ± 6.7)	(42.7 ± 6.6)	(41.9 ± 6.3)

^1^Predicted maximal heart rates =209.6 - 0.67 x age (in years)^29^.

^2^Data were presented in mean ± S.D.

**Table 4:** Blood O_2_ saturation (%SpO_2_) before and immediately following the exercise test^1^.

**MALE**	**%SpO**_**2**_ **in quietness**	**%SpO**_**2**_ **at 30**^**th**^ **sec post-test**	**%SpO**_**2**_ **at 1**^**st**^ **min post-test**	**%SpO**_**2**_ **at 2**^**nd**^ **min post-test**	**%SpO**_**2**_ **at 3**^**rd**^ **min post-test**	**%SpO**_**2**_ **at 4**^**th**^ **min post-test**	**%SpO**_**2**_ **at 5**^**th**^ **min post-test**
***Non-Mi.III (46)***	98.6 ± 0.7	97.7 ± 1.9	98.1 ± 1.3	98.6 ± 0.6	98.8 ± 0.5	98.7 ± 0.5	98.7 ± 0.5
***Mi.III (24)***	99.0 ± 0.0	97.7 ± 1.2	98.3 ± 0.8	98.7 ± 1.3	98.5 ± 1.3	98.7 ± 0.6	98.8 ± 0.6
**FEMALE**							
***Non-Mi.III (63)***	98.8 ± 0.5	97.7 ± 2.3	98.7 ± 0.6	98.8 ± 0.5	98.8 ± 0.4	98.8 ± 0.5	98.8 ± 0.4
***Mi.III (46)***	99.0 ± 0.2	98.3 ± 0.9	98.7 ± 0.7	98.9 ± 0.4	98.9 ± 0.3	98.9 ± 0.3	98.8 ± 0.4

^1^Data were presented in mean ± S.D.

**Table 5:** Blood gas and lactate immediately before and after the stepping test.

		**mean**	**SD**	**mean**	**SD**	***Significance***^3^
		Non-Mi.III male (20)^1^		Mi.III male (10)		
**venous CO**_**2**_ **(mmHg)**	before	55.9	7.8	57.5	7.0	*n.s.*
	after	53.2	7.5	57.8	5.9	*n.s.*
	Difference^2^	−2.69	4.80	0.30	2.91	*P < 0.05
**pH**	before	7.36	0.04	7.35	0.03	*n.s.*
	after	7.34	0.03	7.33	0.04	*n.s.*
	difference	−0.02	0.03	−0.02	0.03	*n.s.*
**HCO**_**3**_^**-**^**act (mM)**	before	30.9	2.7	31.8	2.5	*n.s.*
	after	28.4	3.6	29.7	2.2	*n.s.*
	difference	−2.42	2.02	−2.07	0.77	*n.s.*
**base excess (mM)**	before	4.61	1.77	4.76	2.11	*n.s.*
	after	2.02	2.97	2.40	2.07	*n.s.*
	difference	−2.59	2.15	−2.36	0.72	*n.s.*
**lactate (mg/dL)**	before	12.5	3.5	14.6	5.2	*n.s.*
	after	27.6	14.5	32.5	11.0	*n.s.*
	difference	15.2	14.9	18.0	7.6	*n.s.*

^1^The number inside parentheses indicated the number of blood test subjects.

^2^“Difference” referred to the change of the measured values due to stepping for individual subjects; the difference was one’s post-exercise value subtracted by his pre-exercise value. Shown here were mean±S.D. for each parameter.

^3^To test if the pre-exercise measurements alone (or the post-exercise measurements alone) were significantly different between non-Mi.III and Mi.III groups, *t*-test was used. Another statistical method—ANCOVA was used to assess differences between the pre-exercise and the post-excise measurements for each subject, and to determine whether Mi.III significantly affected the parameter following exercise. “*n.s.*”, not significant.

## References

[b1] Lomas-Francis C. Miltenberger phenotypes are glycophorin variants: a review. ISBT Science Series 6, 296–301 (2011).

[b2] Daniels G. Human blood groups: Geoff Daniels; foreword to first edition by Ruth Sanger. 3rd edn, (John Wiley & Sons, 2013).

[b3] Tippett P. *et al.* The Miltenberger subsystem: is it obsolescent? Transfus Med. Rev. 6, 170–182 (1992). 1498463 10.1016/s0887-7963(92)70167-9

[b4] Race R. R. & Sanger R. Blood groups in man. 6th edn, (Blackwell Scientific Publications, 1975).

[b5] Broadberry R. E. & Lin M. The distribution of the MiIII (Gp.Mur) phenotype among the population of Taiwan. Transfus Med. 6, 145–148 (1996). 8809963 10.1046/j.1365-3148.1996.d01-64.x

[b6] Issitt P. D. Applied blood group serology. 3rd edn, (Montgomery Scientific Publications, 1985).

[b7] Hsu K. *et al.* Assessing the frequencies of GP.Mur (Mi.III) in several Southeast Asian populations by PCR typing. Transfusion and apheresis science : official journal of the World Apheresis Association : official journal of the European Society for Haemapheresis 49, 370–371, doi:10.1016/j.transci.2013.05.011 (2013). 23756267

[b8] Lin M. & Broadberry R. E. Immunohematology in Taiwan. Transfus Med. Rev. 12, 56–72 (1998). 9460191 10.1016/s0887-7963(98)80090-4

[b9] Reithmeier R. A. A membrane metabolon linking carbonic anhydrase with chloride/bicarbonate anion exchangers. Blood Cells Mol. Dis. 27, 85–89, doi:10.1006/bcmd.2000.0353 (2001). 11358366

[b10] Gorman R. B., McKenzie D. K. & Gandevia S. C. Task failure, breathing discomfort and CO2 accumulation without fatigue during inspiratory resistive loading in humans. Respiration physiology 115, 273–286 (1999). 10424357 10.1016/s0034-5687(99)00010-9

[b11] Shikakogi K. *Taitō-chō kannai shisatsu fukumeisho*. (Seibun Shuppansha, 1985 (original 1912)).

[b12] Aoki S. S. The writing about Taiwan by a Japanese police officer who lived in the indigenous communities of Taiwan. (Nenshosha, 2002).

[b13] Bruce L. J. *et al.* Altered structure and anion transport properties of band 3 (AE1, SLC4A1) in human red cells lacking glycophorin A. J. Biol. Chem. 279, 2414–2420, doi:10.1074/jbc.M309826200 (2004). 14604989

[b14] Groves J. D. & Tanner M. J. Glycophorin A facilitates the expression of human band 3-mediated anion transport in Xenopus oocytes. J. Biol. Chem. 267, 22163–22170 (1992). 1385395

[b15] Groves J. D. & Tanner M. J. The effects of glycophorin A on the expression of the human red cell anion transporter (band 3) in Xenopus oocytes. J. Membr. Biol. 140, 81–88 (1994). 8051695 10.1007/BF00234488

[b16] Auffray I. *et al.* Glycophorin A dimerization and band 3 interaction during erythroid membrane biogenesis: *in vivo* studies in human glycophorin A transgenic mice. Blood 97, 2872–2878 (2001). 11313283 10.1182/blood.v97.9.2872

[b17] Blumenfeld O. O. & Huang C. H. Molecular genetics of the glycophorin gene family, the antigens for MNSs blood groups: multiple gene rearrangements and modulation of splice site usage result in extensive diversification. Hum Mutat. 6, 199–209, doi:10.1002/humu.1380060302 (1995). 8535438

[b18] Hsu K. *et al.* Miltenberger blood group antigen type III (Mi.III) enhances the expression of band 3. Blood 114, 1919–1928, doi:10.1182/blood-2008-12-195180 (2009). 19564639 PMC2738576

[b19] Huang C. H. & Blumenfeld O. O. Molecular genetics of human erythrocyte MiIII and MiVI glycophorins. Use of a pseudoexon in construction of two delta-alpha-delta hybrid genes resulting in antigenic diversification. J. Biol. Chem. 266, 7248–7255 (1991). 2016325

[b20] Hsu K. Physiological Implications of Miltenberger blood group antigen subtype III (Mi.III). ISBT Science Series 6, 302–305 (2011).

[b21] Panay I. D. *Taiwan Indigenous Peoples- Ami Tribe*,< http://www.tacp.gov.tw/tacpeng/home02_3.aspx?ID=$3051&IDK=2&EXEC=L>(Date of access: 08/30/2014).

[b22] Kuo H.-M. Study of excellent indigenous people in Taiwan participating track and field games (1946 - 2006) M.S. thesis, National Taitung University, (2007).

[b23] Wu Y. H., CH. Research on the performance of aboriginal athletes in Taiwan track and field competitions. Taiwan Sports 102, 21–29 (1999).

[b24] Salhany J. M. & Schopfer L. M. Kinetic mechanism of DIDS binding to band 3 (AE1) in human erythrocyte membranes. Blood Cells Mol. Dis. 27, 844–849, doi:10.1006/bcmd.2001.0458 (2001). 11783947

[b25] Hsu K., Lin Y. C., Lee T. Y. & Lin M. Miltenberger blood group antigen subtype III (Mi.III) supports Wr(b) expression. Vox Sang 100, 389–394, doi:10.1111/j.1423-0410.2010.01436.x (2011). 21029112

[b26] Education, M. o. *The norm charts of cardiorespiratory endurance indices for Taiwanese populations*, < http://www.fitness.org.tw/model07.php>(2013) (Date of access: 08/30/2014).

[b27] West J. B. Respiratory physiology: the essentials. 7th edn, (Lippincott Williams & Wilkins, 2005).

[b28] Hashimoto H. Higashi Taiwan. (Nangoku Shuppan Kyōkai, 1922).

[b29] Santo A. S. & Golding L. A. Predicting maximum oxygen uptake from a modified 3-minute step test. Research quarterly for exercise and sport 74, 110–115 (2003). 12659482 10.1080/02701367.2003.10609070

[b30] McArdle W. D., Katch F. I. & Katch V. L. Exercise physiology: nutrition, energy, and human performance. 7th edn, (Wolters Kluwer/Lippincott Williams & Wilkins Health, 2010).

[b31] American College of Sports Medicine Position Stand. The recommended quantity and quality of exercise for developing and maintaining cardiorespiratory and muscular fitness, and flexibility in healthy adults. Medicine and science in sports and exercise 30, 975–991 (1998). 9624661 10.1097/00005768-199806000-00032

[b32] McKenna M. J. *et al.* Enhanced pulmonary and active skeletal muscle gas exchange during intense exercise after sprint training in men. J. Physiol. 501 (Pt 3), 703–716 (1997). 9218229 10.1111/j.1469-7793.1997.703bm.x PMC1159470

[b33] Peronnet F. & Aguilaniu B. Lactic acid buffering, nonmetabolic CO2 and exercise hyperventilation: a critical reappraisal. Respiratory physiology & neurobiology 150, 4–18, doi:10.1016/j.resp.2005.04.005 (2006). 15890562

[b34] Lai Y.-C. Misakoliay Kiso Anini Haw?Have you worked as a coolie today?─labor output of Amis in Taitung Prefecture during Japanese colonial period. (Eastern Taiwan Study Association, 2013).

[b35] Hsu K. *et al.* A direct blood polymerase chain reaction approach for the determination of GP.Mur (Mi.III) and other Hil+Miltenberger glycophorin variants. Transfusion 53, 962–971, doi:10.1111/j.1537-2995.2012.03861.x (2013). 22924868

[b36] Salhany J. M., Cordes K. A. & Sloan R. L. Mechanism of band 3 dimer dissociation during incubation of erythrocyte membranes at 37 degrees C. Biochem. J. 345 **Pt 1**, 33–41 (2000). 10600636 PMC1220727

[b37] Education, M. o. *The 3-minute stepping fitness test*,< http://www.fitness.org.tw/measure06.php>(2013)(Date of access: 08/30/2014).

[b38] Devine B. Gentamicin therapy. Drug Intelligence & Clinical Pharmacy 8, 650–655 (1974).

